# Supplementary material for: Solar-Driven Interfacial Evaporation Using Bumpy Gold Nanoshell Films with Controlled Shell Thickness
Source: Int J Mol Sci. 2025 Jun 26;26(13):6160. doi: 10.3390/ijms26136160 (PMC12249822; doi:10.3390/ijms26136160)
Supplement: Supplementary file 1 [file ijms-26-06160-s001.zip › ijms-3646089-supplementary-done.pdf]

International Journal of *Molecular Sciences*

MDPI

Solar-Driven Interfacial Evaporation Using Bumpy Gold Nanoshell Films with Controlled Shell Thickness

Yoon-Hee Kim [1], Hye-Seong Cho [1], Kwanghee Yoo [1], Cho-Hee Yang [1], Sung-Kyu Lee [1], Homan Kang [2] and Bong-Hyun Jun [1,*]

[1] Department of Bioscience and Biotechnology, Konkuk University, Seoul 05029, Republic of Korea; yoonhees@konkuk.ac.kr (Y.-H.K.); joh0302@konkuk.ac.kr (H.-S.C.); heu1997@konkuk.ac.kr (K.Y.); vltizk0052@konkuk.ac.kr (C.-H.Y.); sklee0504@konkuk.ac.kr (S.-K.L.)

[2] Gordon Center for Medical Imaging, Department of Radiology, Massachusetts General Hospital and Harvard Medical School, Boston, MA 02114, USA; hkang7@mgh.harvard.edu

* Correspondence: bjun@konkuk.ac.kr

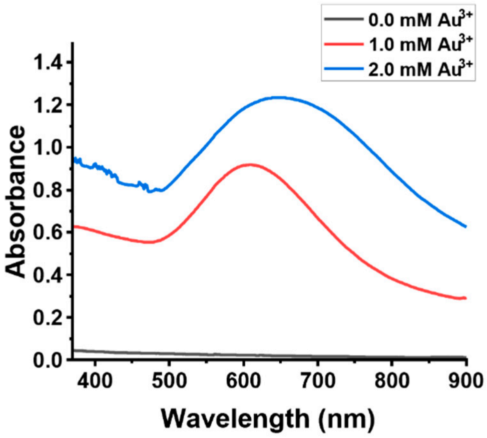

Figure S1. Experimental absorption spectra of bumpy gold nanoshell grown with 0 (black), 1.0 (red), and 2.0 mM Au^{3+} (blue).

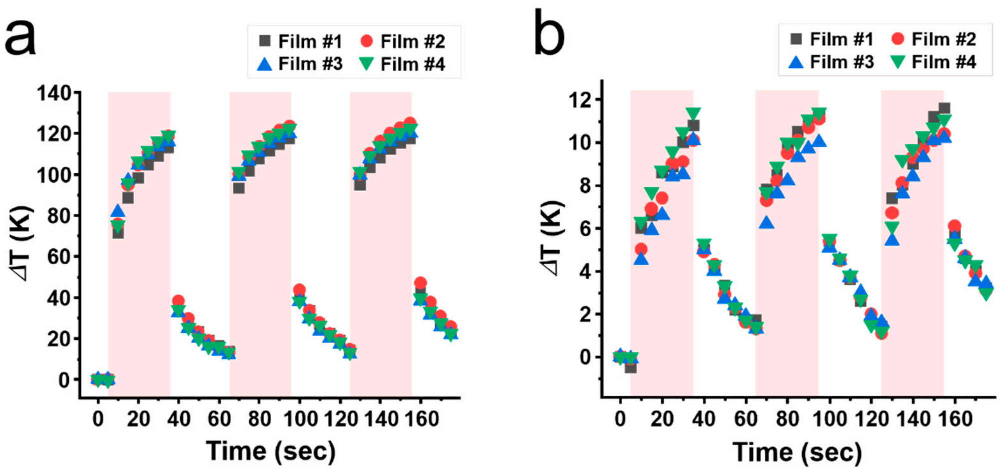

Figure S2. Photothermal effect of bumpy gold nanoshell films (independently fabricated on cellulose filter) under repeated irradiation by a (a) 980 nm laser and (b) an ASTM E 927-05 solar simulator (Newport) operating at AM1.5G.

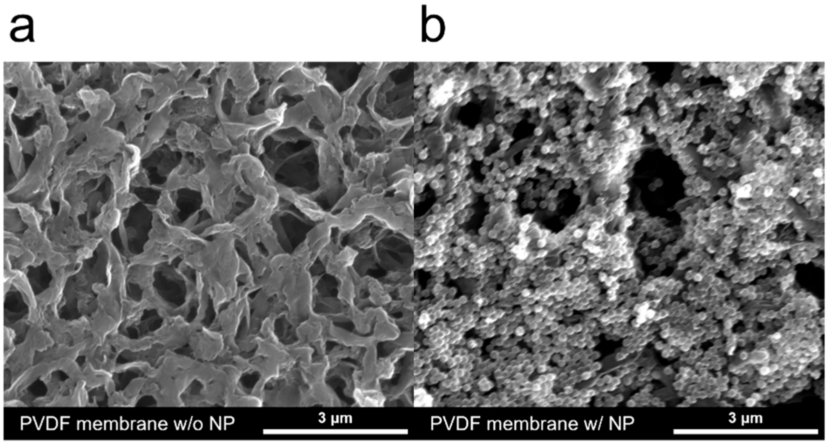

Figure S3. Scanning electron microscopy (SEM) image of (**a**) pristine PVDF membrane and (**b**) bumpy gold nanoshell film-deposited PVDF membrane. The image was captured by Nova Nano-SEM 200 (FEI) operating with an acceleration voltage of 20 kV.
